# Simultaneous Removal of the Freshwater Bloom-Forming Cyanobacterium *Microcystis* and Cyanotoxin Microcystins via Combined Use of Algicidal Bacterial Filtrate and the Microcystin-Degrading Enzymatic Agent, MlrA

**DOI:** 10.3390/microorganisms9081594

**Published:** 2021-07-27

**Authors:** Suqin Wang, Siyu Yang, Jun Zuo, Chenlin Hu, Lirong Song, Nanqin Gan, Peng Chen

**Affiliations:** 1 State Key Laboratory of Fresh Water Ecology and Biotechnology, Institute of Hydrobiology, Chinese Academy of Sciences, Wuhan 430072, China; suqinwanghn@outlook.com (S.W.); yangsiyu@ihb.ac.cn (S.Y.); jzuo@iue.ac.cn (J.Z.); lrsong@ihb.ac.cn (L.S.); 2 Changde Research Center for Agricultural Biomacromolecule, Hunan Key Laboratory for Health Aquaculture and Product Processing in Dongting Lake Area, Hunan University of Arts and Science, Changde 415000, China; 3 University of Chinese Academy of Sciences, Beijing 100049, China; 4 Fujian Key Laboratory of Watershed Ecology, Key Laboratory of Urban Environment and Health, Institute of Urban Environment, Chinese Academy of Sciences, Xiamen 361021, China; 5 College of Pharmacy, University of Houston, Calhoun Road 4849, Houston, TX 77204, USA; chu7@uh.edu; 6 Changde Win-Win Environmental Consulting Service CO., LTD., Changde 415000, China; blackball@yeah.net

**Keywords:** biological removal, *Microcystis* blooms, algicidal bacteria, microcystin release, MlrA enzyme

## Abstract

Freshwater cyanobacterial blooms (e.g., *Microcystis* blooms) constitute a major global environmental problem because of their risks to public health and aquatic ecological systems. Current physicochemical treatments of toxic cyanobacteria cause the significant release of cyanotoxin microcystins from damaged cells. Biological control is a promising eco-friendly technology to manage harmful cyanobacteria and cyanotoxins. Here, we demonstrated an efficient biological control strategy at the laboratory scale to simultaneously remove *Microcystis* and microcystins via the combined use of the algicidal bacterial filtrate and the microcystin-degrading enzymatic agent. The algicidal indigenous bacterium *Paenibacillus* sp. SJ-73 was isolated from the sediment of northern Lake Taihu, China, and the microcystin-degrading enzymatic agent (MlrA) was prepared via the heterologous expression of the *mlrA* gene in the indigenous microcystin-degrading bacterium *Sphingopyxis* sp. HW isolated from Lake Taihu. The single use of a fermentation filtrate (5%, *v*/*v*) of *Paenibacillus* sp. SJ-73 for seven days removed the unicellular *Microcystis aeruginosa* PCC 7806 and the native colonial *Microcystis* strain TH1701 in Lake Taihu by 84% and 92%, respectively, whereas the single use of MlrA removed 85% of microcystins. Used in combination, the fermentation filtrate and MlrA removed *Microcystis* TH1701 and microcystins by 92% and 79%, respectively. The present biological control thus provides an important technical basis for the further development of safe, efficient, and effective measures to manage *Microcystis* blooms and microcystins in natural waterbodies.

## 1. Introduction

Cyanobacterial blooms can have long-term negative impacts on water-quality and ecosystem health, such as increasing water turbidity, diminishing dissolved oxygen, smothering fish, producing unpleasant odor compounds, and excreting various cyanotoxins [1,2]. The control of harmful cyanobacterial blooms might become more challenging in the future, because it is widely confirmed that cyanobacterial blooms are gradually increasing in frequency, magnitude, and duration on a global scale owing to progressive eutrophication, rising atmospheric CO_2_ levels, and global warming [3,4]. Increasing attention has been paid to *Microcystis* spp. because of their extreme abundance, frequent bloom occurrence, and capacity to produce microcystins (MCs), which are the most commonly reported and problematic cyanotoxins worldwide [5]. The World Health Organization established a guideline of 1 μg/L for the highest acceptable concentration of microcystin LR (MC-LR) in drinking water [6].

The *Microcystis* spp. and the resultant contamination by MCs have posed a serious threat to the ecological environment and to public health [7]. Therefore, the development of efficient and sustainable methods of removing *Microcystis* spp. blooms and MCs has become an urgent issue. Various physical and chemical techniques were developed to control and mitigate cyanobacterial blooms, including flocculation [8], ultrasonic waves [9], and the application of toxic chemicals, such as copper sulfate [10]. However, these techniques have certain drawbacks in terms of high energy cost, low efficacy, and side effects. To address these issues, biological strategies employing microbes have attracted increasing attention in recent decades [11]. Algicidal bacteria are one of the key biological agents that are frequently involved in the dramatic decline and termination of cyanobacterial blooms in the field [11,12]. A variety of algicidal bacteria were isolated from diverse natural environments, which belong to the genera *Flavobacterium*, *Pseudomonas*, *Bacillus*, *Pseudoalteromonas*, *Alteromonas*, and *Streptomyces* [13]. They can inhibit or kill algae either by directly attacking the algal cells, for which physical contact is required, or indirectly by competing for nutrients or excreting algicidal compounds [13,14]. Algicidal substances comprise of alkaloids [15], enzymes [16], peptides, amino acids derivatives [17], polyketides [18], and terpenes [19]. 

The lysis of cyanobacteria during the bloom decline period can result in the large release of cyanotoxins into the surrounding water. A long-term assessment of MC concentrations in Lake Taihu showed that the dissolved MCs in the water column ranged from 2.20 μg/L to 16.23 μg/L during 2006–2013, with a mean of 4.90 μg/L in Zhushan Bay [20], which exceeded the safety threshold value by 5-fold. The cyclic structure of MCs exhibits marked resistant to high temperature, extreme pH, and sunlight in the natural environment, with a reported environmental half-life of ten weeks [21]. Conventional water treatment technologies, such as membrane filtration and coagulation sedimentation, cannot effectively remove and detoxify MCs; therefore, more expensive methods, such as photocatalysis, activated carbon, or oxidation processes might be required, which limits their sustainable application. A series of studies confirmed that some indigenous bacteria associated with *Microcystis* colonies, or distributed in natural water columns and sediments, display degradation activity against MCs, which might be ideal materials for MC biological treatment [22,23]. To date, a number of MC-degrading bacteria have been isolated, with the majority of the groups belonging to the *Sphingomonas* and *Sphigopyxis* genera [24]. 

The genetic and enzymatic mechanism of the Microcystinase (Mlr)-dependent pathway for MC biodegradation was fully described, in which MC is degraded sequentially into linearized MC, tetrapeptides, Adda, phenylacetic acid, and ultimately, CO_2_, via a series of specific enzymes encoded by *mlr* and neighboring *paa* gene clusters [25,26,27]. The *mlr*-dependent degradative activity of indigenous bacteria is under strict control and is affected by multiple factors [28,29,30]. Therefore, the direct modulation of wild MC-degraders in practical applications remains a challenge. Heterologous expression of *mlr* genes was developed as a powerful approach to elucidate the enzymatic mechanism of the *mlr*-pathway. Additionally, it offers an opportunity to boost the production of the target enzyme by using a fast-growing heterologous producer. Microcystinase A (MlrA) is the first key enzyme involved in hydrolyzing MC, producing a linearized MC with 160-fold decrease in toxicity [31]. To date, heterologous expression of MlrA has been developed in the heterotrophic host *Escherichia coli* [32] and a newly reported photoautotrophic host, *Synechocystis* sp. PCC 6803 [33].

The majority of water remediation strategies have only focused on mitigating cyanobacteria blooms or the resultant toxins, not both. In the present study, the combined application of algicidal bacteria and the MC biodegradation agent MlrA was proposed to remove excess cyanobacteria and MCs simultaneously. The algae-lysing performance of the strain *Paenibacillus* sp. SJ-73 and the desirable conditions for heterologously expressing MlrA will be characterized. Moreover, the efficiency of the co-application of the algicidal bacteria and MlrA will be evaluated in terms of the cyanobacteria biomass removal rate and the MC degradation rate. 

## 2. Materials and Methods

### 2.1. Microorganisms and Culture Conditions 

The bacterial strain *Sphingopyxis* sp. HW was previously isolated from the sediment of Lake Taihu, China [34]. *Pseudomonas aeruginosa* (CCTCC-AB91095) was purchased from the China Center for Type Culture Collection (CCTCC, Wuhan, China) as a negative control (non-algae-lysing bacterium). All bacteria were inoculated into Luria-Bertani (LB) medium or Gause’s medium, and grown at 30 °C, with shaking at 200 rpm, for 24–48 h. Axenic strains *Microcystis aeruginosa* (PCC 7806), *Anabaena* sp. (PCC 7120), and non-axenic strains *Aphanizomenon flos-aquae* (FACHB 1168), *Chlorella sorokiniana* (FACHB 26), and *Tetradesmus obliquus* (FACHB 416) were obtained from the Freshwater Algae Culture Collection at the Institute of Hydrobiology (FACHB, Wuhan, China). Colonial *M. aeruginosa* TH1701 was isolated from Lake Taihu. The algae were cultivated in a BG11 medium [35] at 25 °C with a light-dark cycle of 12:12 h under a light intensity of 30 μmol photons m^−2^ s^−1^.

### 2.2. Isolation and Characterization of Algicidal Bacteria

#### 2.2.1. Screening for Algicidal Bacteria Strains

One gram of sediment sampled from Meiliang Bay, Lake Taihu, was suspended in 10 mL of sterilized deionized water (dH_2_O), spread onto LB plates, and incubated at 30 °C for 24 h. A single colony was randomly picked to screen potential algicidal bacteria by observing their effects on the growth of *M. aeruginosa* PCC 7806 in 24-well plates. Thereafter, cultures of potential algicidal bacteria (5%, *v*/*v*) were added into an axenic culture of *M. aeruginosa* PCC 7806 to measure their algicidal efficiency. The algicidal efficiency was calculated using the formula:

Algicidal efficiency (%) = [(C_0_ − C)/C_0_] × 100%

where C_0_ was the chlorophyll *a* (Chl-*a*) concentration at the initial stage, while C was the Chl-*a* concentration at the end of the experiment. The concentration of Chl-*a* was measured according to standard methods [36]. Bacterial cultures of *Pseudomonas aeruginosa* (CCTCC-AB91095) were used as the negative control. The strain with the strongest algicidal efficiency was isolated and prepared for subsequent experiments. 

#### 2.2.2. Morphology and Molecular Identification of Algicidal Bacterial Strain SJ-73

Strain SJ-73 was identified by its morphological characteristics and 16S rDNA sequence. Cells of strain SJ-73 were collected by centrifugation at 4000× *g* at 25 °C for 10 min, and washed with 0.1 M phosphate-buffered saline (PBS) twice. The washed cells were added into 2.5% (*v*/*v*) glutaraldehyde and fixed at 4 °C overnight. The cells were then dehydrated through a graded series of ethanol (30%, 50%, 70%, 90% and 100%, *v*/*v*) for 10 min and then freeze-dried. Finally, the cells were coated with a 20 nm-thick layer of gold and palladium, and their morphological features were observed under a scanning electron microscope (SEM) (Hitachi-S4800, Hitachi, Tokyo, Japan) [37].

The 16S rDNA sequence of strain SJ-73 was sequenced and compared using BLAST with similar 16S rDNA sequences extracted from GenBank. A phylogenetic tree was constructed by the neighbor-joining method using the software molecular evolutionary genetics analysis version 7.0 (MEGA 7.0) [38]. The bacterial concentrations throughout the experiments were determined using the colony-forming units procedure [39] and measured using the optical density at 600 nm (OD_600_).

#### 2.2.3. Algicidal Pathway and Host Specificity of Strain SJ-73

The bacterial culture was centrifuged for 10 min at 4000× *g* to collect two different fractions, including the cells and the cell-free supernatant. The filtrate was collected for further experiments by filtering the cell-free supernatant through a 0.22 µm fast flow and low binding Millipore express PES membrane (Merck Millipore Ltd., Dublin, Ireland). To determine the direct or indirect algicidal pathway of SJ-73 against *M*. *aeruginosa*, the filtrate and the isolated cells were separately added into the algal culture of *M. aeruginosa* PCC 7806 at 5% (*v*/*v*) to assess their algicidal efficiency by measuring Chl-*a.*

To investigate the algicidal host specificity against the different bloom-forming cyanobacteria species, *M*. *aeruginosa* (PCC 7806), *Ana.* sp. (PCC 7120) and *Aph. flos-aquae* (FACHB 1168) were co-cultivated with SJ-73 filtrate for 7 days. In addition, two green algae, *C. sorokiniana* (FACHB 26) and *T.* *obliquus* (FACHB 416) were tested as well. The algicidal efficiency against these species were measured on the first and seventh day based on the Chl-*a* contents.

#### 2.2.4. Algicidal Effects on the Growth of Unicellular and Colonial *M. aeruginosa*

The strain SJ-73 and *Pseudomonas aeruginosa* AB91095 were inoculated into a liquid LB medium at 30 °C, with shaking at 200 rpm for 24 h. Then, the bacterial cultures were centrifuged for 10 min at 4000× *g* and filtered through a 0.22 μm membrane to collect filtrates. For unicellular PCC 7806, 6 mL of SJ-73 or AB91095 filtrates were added into 120 mL of *M. aeruginosa* PCC 7806 in a conical flask. For colonial *M. aeruginosa* TH1701, 6 mL of SJ-73 filtrate or Gause’s medium were mixed with 120 mL colonial *M. aeruginosa* TH1701 cultures. The algicidal effects of the strain SJ-73 on *M. aeruginosa* PCC 7806 and colonial *M. aeruginosa* TH1701 were evaluated using the algicidal efficiency and photosynthetic activity. 

The Chl-*a* concentrations were measured every two days and the photosynthetic activities parameter (*Fv*/*Fm*) was tested every day using a WATER-PAM (WATER-ED, Heinz WALZ, Germany) [40].

#### 2.2.5. Effects on Cell Morphology 

To further investigate the process of algal cell lysis under treatment by strain SJ-73, the cells were observed under an SEM and a transmission electron microscope (TEM) (Hitachi-7700, Hitachi, Tokyo, Japan). *M. aeruginosa* PCC 7806 cells were harvested by centrifugation at 4000× *g* for 10 min after treatment with the strain SJ-73 filtrate for 0, 3 and 7 days. The collected cells were fixed overnight under 4 °C in 0.1 M PBS (pH 7.4) and dehydrated through a graded series of ethanol (30%, 50%, 70%, 90% and 100%, *v*/*v*) for 10 min. The fixed samples were subjected to SEM and TEM observations according to the standard methods [37].

### 2.3. MlrA Heterologous Expression

#### 2.3.1. Cloning and Heterologous Expression of MlrA

*Sphingopyxis* sp. HW was cultured in an LB medium with 5 ng/L MC-LR at 30 °C for 48 h, and then genomic DNA was extracted using an MP FastDNA SpinDown Kit (MP Biomedicals, LLC, Illkirch-Graffenstaden, France). The *mlrA* gene sequence was acquired by whole genome sequencing (data not published), and the *mlrA* gene was amplified from the whole genomic DNA of *Sphingopyxis* sp. HW using primers mlrA-F (5′-AGGAGAATACCATGCGGGAGTTTGTCAAACAGCGA-3′) and mlrA-R (5′-TGGTGGTGGTGATGATGCGCGTTCGCGCCGGAC-3′). PCR was performed in a total volume of 50 μL, including 25 μL KAPA HiFi Hot Start Ready Mix (KAPA Biosystems. Co., Ltd., Wilmington, MA, USA), 0.8 μL forward and reverse primer, 2 μL DNA samples, and 21.4 μL ddH_2_O. The PCR reaction included initial denaturation at 95 °C for 4 min; followed by 30 cycles of 20 s at 95 °C, 55 °C for 15 s, and 72 °C for 100 s, with a final extension step for 5 min at 72 °C.

The target band was excised from a 1.2% (*m*/*v*) agarose gel and purified using a TIANGEN DNA purification Kit (TIANGEN Biotech. Co., Ltd., Beijing, China) according to the manufacturer’s instructions. Subsequently, the amplicon was ligated into vector pET28H using a ClonExpress^®^Ultra One Step Cloning Kit (Vazyme Biotech. Co., Ltd., Jiangsu, China) to obtain the plasmid, which was transformed into *E. coli* BL21 (DE3) competent cells.

The expression of recombinant MlrA was according to reference [32], with minor modifications. The bacteria and negative control were grown in LB media with 50 μg/mL kanamycin and with shaking at 180 rpm under 30 °C. When the OD_600_ of the cultures reached 0.6, the expression was induced with 0.2 mM isopropyl-*β*-d-thiogalactoside (IPTG, sigma, USA) at 18 °C overnight. The cultures were centrifuged at 4000× *g* and 25 °C for 10 min and re-suspended in PBS (50 mM, pH 7.4) at an equal volume. Then, the samples were treated using a low temperature ultra-high-pressure continuous flow cell disrupter (750 W, HN-mini Juneng Biotech. Co., Ltd., Guangzhou, China) to lyse the cells, and centrifuged at 8000× *g*, 4 °C for 20 min to collect the cell-free extract, which contained MlrA. 

#### 2.3.2. Analysis of MlrA Activity by HPLC-PDA and UPLC-MS/MS

To measure the activity of the expressed MlrA, an equal volume of cell-free extract was mixed with an equal volume of 7.5 mg/L [D-Asp^3^]MC-LR then incubated at 30 °C for 30 min. The mixture samples of MlrA and [D-Asp^3^]MC-LR were collected at 0, 5, 15, and 30 min by centrifuging at 8000× *g* for 20 min before analysis. [D-Asp^3^]MC-LR degradation was assessed by monitoring the decrease in its concentration using high-performance liquid chromatography–photodiode array detection (HPLC–PDA) (Waters 2998-2690/2695, Waters, Milford, MA, USA) [36]. All experiments were conducted in triplicate.

The degradation product sample was used to determine the molecular weight (MW) of the parent [D-Asp^3^]MC-LR and its degradation products using ultraperformance liquid chromatography-tandem mass spectrometry (UPLC-MS/MS) (LC-Q-TOF 1290II/6545B, Agilent, Santa Clara, CA, USA). The drying gas temperature and flow rate were set at 320 °C and 8 L min^−1^, respectively. The mobile phase consisted of a gradient of water (solvent A) and acetonitrile (solvent B), both containing 0.05% formic acid (Sigma, St. Louis, MO, USA). The samples were run with the following linear gradient program: 20% B to 80% B over 8 min, then to 80% B over 2 min, where it was held for 2 min. 

### 2.4. Characterization of MlrA

#### 2.4.1. Preparation of MlrA

Cell cultures were harvested by centrifugation (4000× *g* at 4 °C for 25 min), and washed twice with PBS (50 mM, pH 7.4). The cells were disrupted using a cell disrupter (750 W for 20 min) in an ice bath. After centrifugation, the supernatant was collected as a cell-free extract (CE) containing crude protein. The total protein contents were determined using an enhanced BCA protein assay kit (Beyotime Biotech. Co., Ltd., Jiangsu, China) and the concentration of the MlrA enzyme was detected using a His-tag enzyme-linked immunosorbent assay (ELISA) detection kit (Genscript Biotech. Co., Ltd., Piscataway, NJ, USA). 

#### 2.4.2. The Degradation Efficiency and Characteristics of MlrA 

To analyze the characteristic of MlrA, expression assays were carried out using three parallel experiments. The CE, containing 0.8 mg/L MlrA protein, was added into the test tubes with ddH_2_O, containing 7.5 mg/L [D-Asp^3^]MC-LR, while PBS was used in the control. The degradation efficiency of MlrA treatment was evaluated by the changes in [D-Asp^3^]MC-LR concentrations and was calculated by the formula:

Degradation Efficiency (%) = [(C_0_ − C)/C_0_] × 100%

where C_0_ was the [D-Asp^3^]MC-LR concentration at initial stage, while C was the [D-Asp^3^]MC-LR concentration at the end of experiments. 

To detect the minimum degradable MC concentration, [D-Asp^3^]MC-LR was diluted with double distilled water to different concentrations (0.8 μg/L, 5 μg/L and 50 μg/L), and 200 μL [D-Asp^3^]MC-LR and 200 μL CE dissolved in PBS were mixed for 30 min at 25 °C, with 4 μL trifluoroacetic acid (TFA, Sigma, USA) used to terminate the reaction.

To explore the optimum temperature and pH of the MlrA, reaction mixtures containing 200 μL of [D-Asp^3^]MC-LR and 200 μL CE dissolved in PBS were incubated at different temperatures (10, 15, 20, 25, 30 and 35 °C) and different pH values (6, 7, 8 and 9) for 30 min. Then, 4 μL of TFA solution was added to terminate the reactions over 10 min. After centrifugation for 20 min at 8000× *g*, the supernatant was used to determine residual [D-Asp^3^]MC-LR concentrations using HPLC. 

#### 2.4.3. Efficiency of Immobilized and Free MlrA

Sodium alginate was dissolved in sterile water to a final concentration of 3.0%. An equal volume of MlrA was mixed with an equal amount of sodium alginate solution at a 1:1 ratio. Then, the mixture was added into 4% (*m*/*v*) calcium chloride (Sigma, St. Louis, MO, USA) solution and gently stirred at room temperature for 30 min to form immobilized beads. The immobilized beads were washed with sterilized water 3–5 times to remove the calcium chloride on the surface. The immobilization efficiency was calculated according to the following formula:

Immobilization Efficiency (%) = (m_0_ − C × V)/m_0_ × 100%

where m_0_ was the amount of protein at initial stage; C was concentration of remaining protein in solution; and V was the volume of the solution. The immobilized beads were stored at 4 °C and 25 °C, separately. To explore the stability of free MlrA and immobilized MlrA over time, samples were taken out of storage and incubated with 5 μg/mL [D-Asp^3^]MC-LR for 30 min at 25 °C every day. 

### 2.5. Combined Treatment of Algicidal Bacteria and MlrA on M. aeruginosa and MCs

The strain SJ-73 filtrate was collected and filtered through a 0.22 µm fast flow and low binding Millipore express PES membrane. Then, the filtrate (5%, *v*/*v*) was poured into *M. aeruginosa* PCC 7806 and colonial *M. aeruginosa* TH1701 cultures, respectively. At the beginning of experiments, the cell densities of the PCC 7806 culture reached 4.4 × 10^7^ cells/mL and the Chl-*a* concentrations of TH1701 were 500 μg/L. Both were higher than those of cyanobacteria during the harmful cyanobacterial blooms of Lake Taihu. 

For the axenic unicellular strain *M. aeruginosa* PCC 7806, 6 mL of 0.8 mg/L MlrA or PBS (50 mM, pH 7.4) was added into 60 mL of *M. aeruginosa* PCC 7806 cultures treated with strain SJ-73 for 6 days. For the non-axenic colonial strain *M. aeruginosa* TH1701, 6 mL of 0.8 mg/L MlrA was mixed with the SJ-73 filtrate and added to the algal culture at the beginning of the experiment. Then, 0.8 mg/L MlrA was added again on the third day. The concentrations of MCs in all samples were determined using a microcystins ELISA detection kit (Institute of Hydrobiology, Wuhan, China) according to the manufacturer’s instructions. All experiments were conducted in triplicate. 

### 2.6. Statistical Analysis

All experiments were performed in triplicate and presented as means ± standard deviation of the replicates using standard parametric analyses. The significant differences among all the data were evaluated using ANOVA in SPSS 19.0 for windows (IBM Corp., Armonk, NY, USA), with *p* < 0.05 (*) and *p* < 0.01 (**).

## 3. Results

### 3.1. Isolation and Identification of Indigenous Algicidal Bacteria

**Figure 1:**
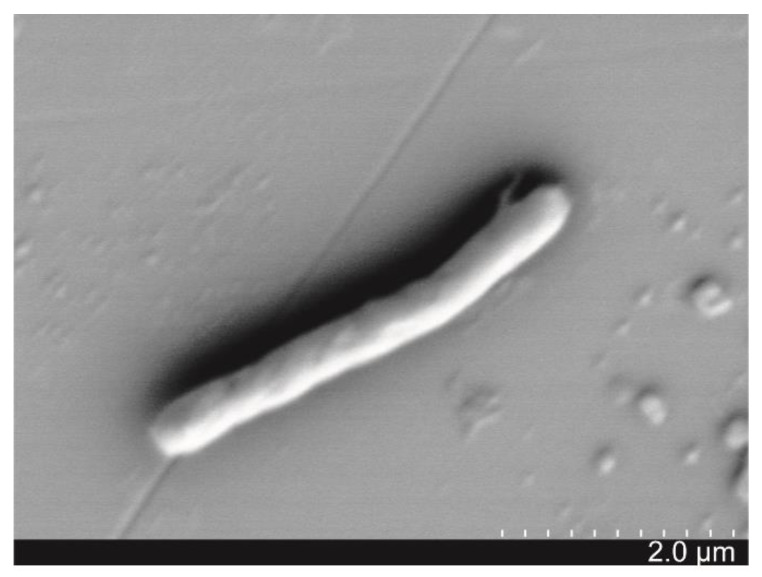
Scanning electron microscopy (SEM) image of morphological characteristics of algicidal bacterium SJ-73.

Bacterium strain SJ-73, which causes the lysis of *M. aeruginosa*, was isolated from the sediment sample of Lake Taihu during a cyanobacterial bloom period. It was a Gram-positive and rod-shaped bacterium (0.3–0.5 μm × 1–3 μm) (Figure 1), which formed white colonies with a smooth surface on Gause’s and LB plates. Phylogenetic analysis based on 16S rDNA sequence revealed that strain SJ-73 was affiliated with the genus *Paenibacillus*, sharing the highest similarity (99.93%) with the *Paenibacillus amylolyticus* strain HSE1 (accession number: MK764982.1) (Figure S1). 

### 3.2. Algicidal Characteristics of Strain SJ-73

#### 3.2.1. Algicidal Model and Spectrum

**Figure 2:**
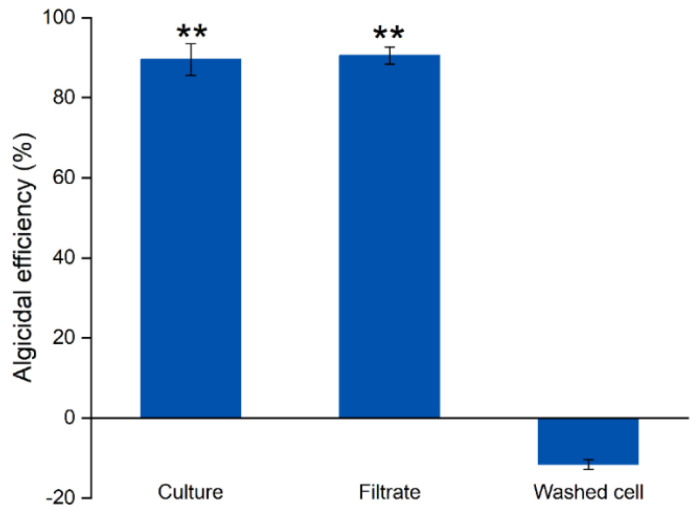
The algicidal efficiency of the culture, filtrate, and washed cell and of *Paenibacillus* sp. SJ-73 against *M. aeruginosa* PCC 7806 after 7 days’ treatment. Bars represent the standard errors of the means for triplicate. Significant differences are shown by asterisks: **, *p* < 0.01.

The algicidal mode of strain SJ-73 was determined by comparing the lytic activity among the bacterial culture, cell-free filtrate, and washed cells against the *Microcystis aeruginosa*. The bacterial culture of strain SJ-73 and the cell-free filtrate exhibited significant inhibition against *Microcystis aeruginosa*, with algicidal rates of 90.48% and 89.51%, respectively (*p* < 0.01), whereas the washed cells of strain SJ-73 exhibited no inhibitory effect (Figure 2). This suggested that strain SJ-73 exerted its algicidal activity mainly by secreting active compound(s).

Strain SJ-73 had a relatively broad algicidal spectrum, the common bloom-forming cyanobacteria species of *Microcystis*, *Anabaena* and *Aphanizomenon* were all susceptible to this bacterium, with algicidal rates of 83.97%, 71.35% and 71.04%, respectively, after 7 days’ treatment with 5% (*v*/*v*) fermentation filtrate of strain SJ-73. By contrast, SJ-73 showed weak inhibitory activity toward unicellular green algae *Chlorella* (3.67%) and *Tetradesmus* (8.03%) (Table 1). 

#### 3.2.2. Algicidal Activity of Strain SJ-73 on Unicellular and Colonial *Microcystis* Strains and the Release of MCs 

**Figure 3:**
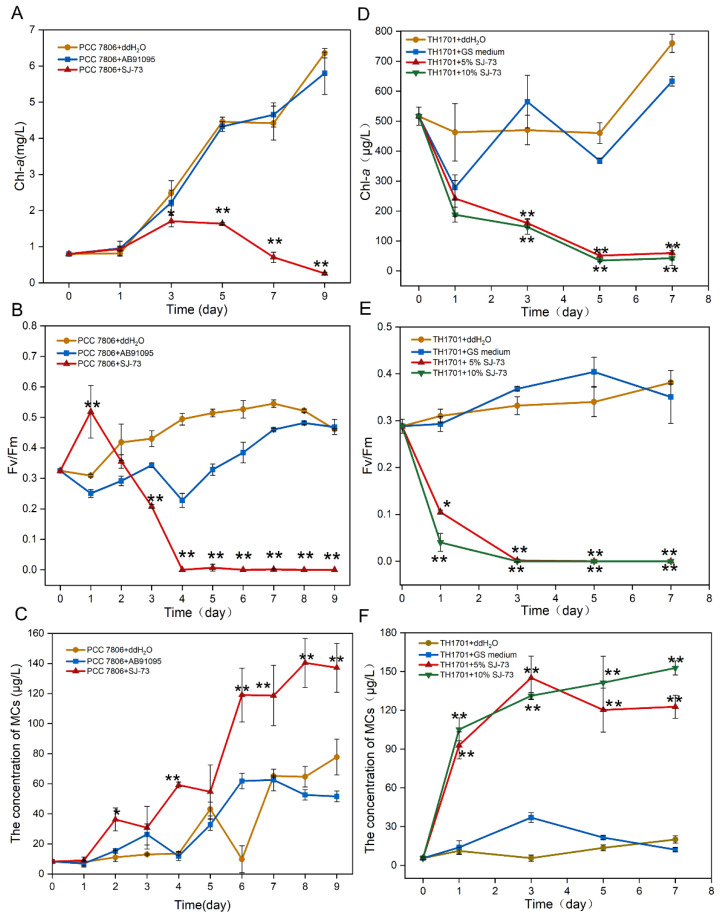
Effects of bacterium SJ-73 on the concentration of Chl-*a* (**A**,**D**), photosynthetic activities (**B**,**E**) and extracellular MCs (**C**,**F**) of unicellular *M. aeruginosa* PCC 7806 and colonial *M. aeruginosa* TH1701 cultures. Bars represent the standard errors of the means for triplicate. Significant differences are shown by asterisks: *, *p* < 0.05; **, *p* < 0.01.

As shown in Figure 3, upon the addition of a cell-free filtrate of SJ-73, for both strains (PCC 7806 and TH1701), the cell growth and photosynthetic activities were significantly suppressed. Meanwhile, a serious release of MCs was observed following cell rupture in the treated groups.

For the axenic unicellular strain *M. aeruginosa* PCC 7806, when 5% (*v*/*v*) cell-free filtrate was added, the obvious decrease in the content of Chl-*a* occurred at day 3, and the algicidal rate of strain SJ-73 against *M. aeruginosa* PCC 7806 reached 95.87% on day 9, while the Chl-*a* content of the control increased by 7.85-fold compared with the initial content (Figure 3A). The maximum quantum yield of PSII (*Fv/Fm*) of *M. aeruginosa* PCC7806 increased on the first day and then dramatically declined from 0.52 to 0 at day 4, whereas the *Fv/Fm* in the control remained around 0.494 (Figure 3B). The addition of filtrate of SJ-73 caused marked MC release, the concentration of extracellular MCs in the treated group steadily increased from 8.31 μg/L to 36.27 μg/L after co-cultivation for 2 days, and the maximum release of MCs (140.40 μg/L) occurred on day 8, while the concentration of MCs in the control group was only half (64.65 μg/L) (Figure 3C).

For the non-axenic colonial strain *M. aeruginosa* TH1701, when two concentrations (5% and 10%, *v*/*v*) of cell-free filtrate were added, the Chl-*a* content of treated *M. aeruginosa* TH1701 decreased dramatically from day 0. The algicidal rates for 5% (*v*/*v*) and 10% (*v*/*v*) treated groups were 47.66% and 59.58%, respectively, at day 1. And reached 92.10% and 94.38% at day 7 (Figure 3D). The *Fv/Fm* of *M. aeruginosa* TH1701 declined from 0.29 to 0.11 and 0.04 within 1 day, and were not detected after day 3 (Figure 3E). The concentration of extracellular MCs in the 5% (*v*/*v*) and 10% (*v*/*v*) treated groups increased markedly; the maximum concentration of MCs reached 122.77 μg/L and 152.84 μg/L at day 7, respectively (Figure 3F). Overall, the algicidal rate was slightly higher in the 10% (*v*/*v*) than in the 5% (*v*/*v*) treated group; however, the 10% (*v*/*v*) treated group displayed more severe MC release during the late period. 

#### 3.2.3. Changes in Cell Morphology

**Figure 4:**
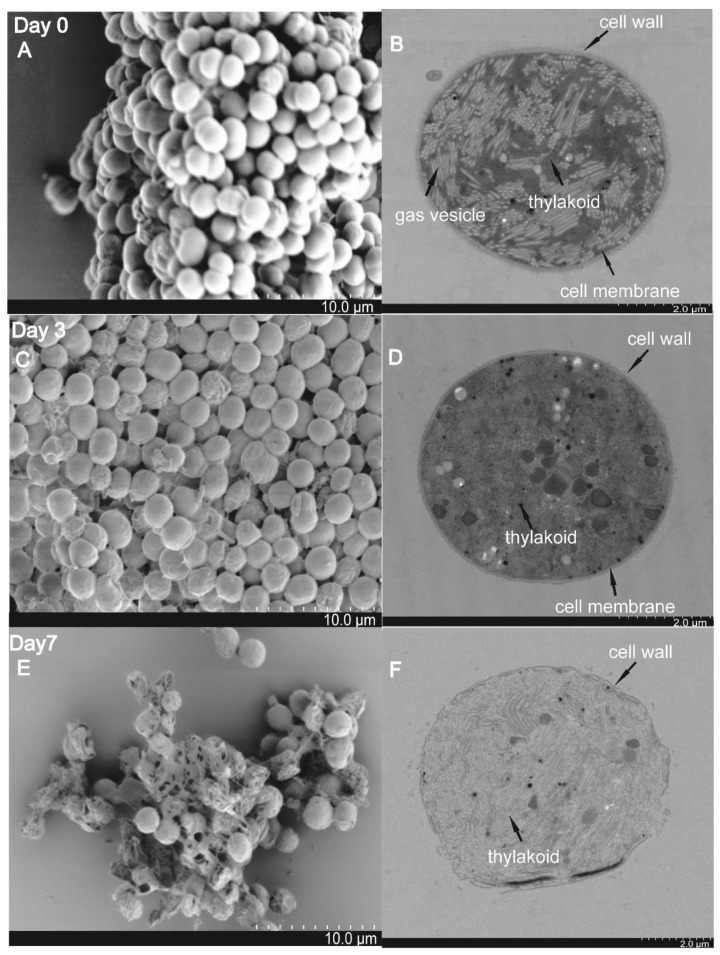
Cell morphology and ultrastructures of *M. aeruginosa* PCC 7806 visualized by scanning electron microscopy (SEM) and transmission electric microscopy (TEM) after treatment with SJ-73 filtrates for 0 days (**A**,**B**), 3 days (**C**,**D**), and 7 days (**E**,**F**).

The changes of the cell surface and ultrastructure of treated *M. aeruginosa* PCC7806 cells were recorded using an SEM and TEM, and samples were collected at day 0, day 3, and day 7. On day 0, the intensive and intact microsphere cells were observed: the cells were plump and rounded with smooth surfaces (Figure 4A); thylakoids and gas vesicles were stacked in an orderly and tight manner in the cytoplasm, and the cell wall was distinct (Figure 4B). On day 3, part of the cells had shrunk, and deformed cells with rough surfaces were observed (Figure 4C); the thylakoids and gas vesicles were disrupted, the cytoplasm was disordered, and the cell wall was obscure, but still maintained the basic shape (Figure 4D). On day 7, the integrity of the cells was severely damaged, (Figure 4E), and the cell wall appeared disrupted; thylakoids and gas vesicles could not be distinguished; and cytoplasmic vacuolation and cytoclasis were evident (Figure 4F). These results suggested that the algicidal compound secreted by SJ-73 could destroy the cell membrane system and cell wall.

### 3.3. Heterologous Expression of MlrA and Its Characterization

#### 3.3.1. Expression of MlrA

**Figure 5:**
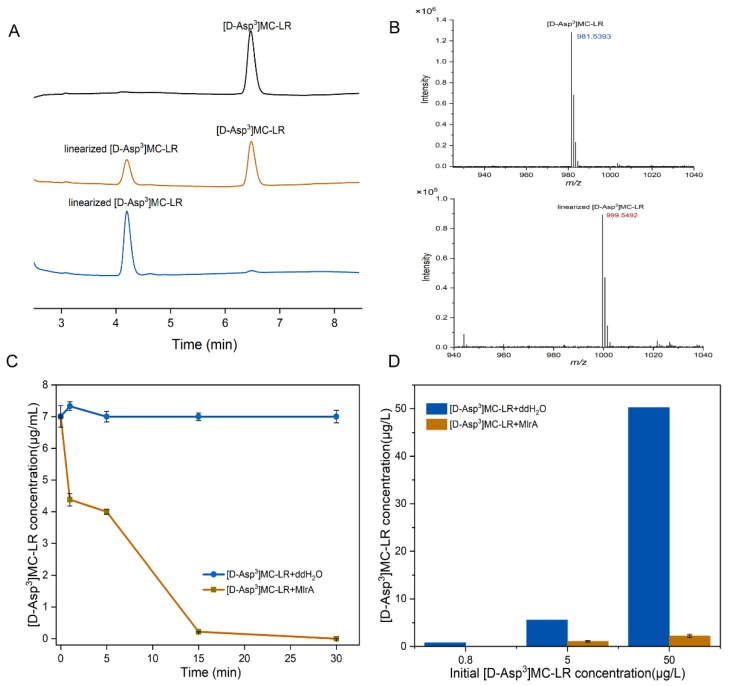
(**A**) HPLC chromatographic presentation of the MlrA activity of the cell-free extracts of recombinant pET28h-*mlrA* BL21(DE3). Peaks with retention time of 6.5 min and 4.2 min correspond to [D-Asp^3^]MC-LR and linearized [D-Asp^3^]MC-LR, respectively. (**B**) MS spectra of [D-Asp^3^]MC-LR (*m*/*z*: 981.5393) and linearized [D-Asp^3^]MC-LR (*m*/*z*: 999.5492); (**C**) degradation curve of [D-Asp^3^] MC-LR by 0.8 mg/L MlrA (30 °C, pH = 7), and (**D**) changes in different concentrations of [D-Asp^3^]MC-LR concentration treated with 0.8 mg/L MlrA. Bars represent the standard errors of the means for triplicate.

The degradation activity of the crude MlrA enzyme from recombinant *E. coli* cells was monitored using HPLC-PDA. As shown in Figure 5A, [D-Asp^3^]MC-LR (retention time 6.5 min) was degraded to a new product with a retention time of 4.2 min. The maximum UV absorbance (λmax) of the new product was 235.4 nm compared with a λmax of 237.8 nm for the parent [D-Asp^3^]MC-LR. The UPLC-MS-MS analysis of the new product revealed an ion at *m*/*z* 999.5, corresponding to the molecular weight of linear [D-Asp^3^]MC-LR (Figure 5B). The recombinant MlrA showed a high degradation efficiency, being able to hydrolyze [D-Asp^3^]MC-LR from an initial concentration of 7.50 mg/L to 0.16 mg/L within 5 min, and almost totally degraded [D-Asp^3^]MC-LR after 15 min (Figure 5C). The pseudo-first-order rate constant was up to 9.36 h^−1^. The minimum degradable MC concentration was below 0.8 μg/L (Figure 5D) as assessed using UPLC-MS detection.

#### 3.3.2. Degradation Efficiencies of MlrA under Different pH and Temperature Conditions

Single factor experiments were performed (results shown in Figure S2), and MlrA degradation efficiencies fluctuated in the pH range of 6.0–9.0. The optimal pH of the reaction was 8.0 and the degradation ratio was 80.21%, while the ratio reduced to 64.59% when the pH decreased to 6.0 (Figure S2A). The optimal temperature was 30 °C, at which degradation efficiency reached 93.43%; however, the degradation efficiency of MlrA decreased to below 55.01% at 10 °C, while it remained above 88.0% when the temperature increased to 35 °C (Figure S2B). 

#### 3.3.3. Sustainability of Free MlrA and Immobilized MlrA

Figure S3 shows the sustainability of free MlrA and immobilized MlrA. The degradation efficiency of free MlrA at 4 °C and 25 °C reached 84.75% and 85.95%, respectively, after 48 h. For immobilized MlrA, the activity reduced to 1.24% of the initial activity after 48 h at 25 °C, but could still hydrolyze [D-Asp^3^]MC-LR with a degradation ratio of 59.32% when kept under 4 °C for 48 h. 

### 3.4. The Combined Treatment of Algicidal Bacteria and MlrA Enzymes to Remove Toxic Microcystis and Resultant MC

#### 3.4.1. The Activity of MlrA in SJ-73 Treated Unicellular *M. aeruginosa* Cultures

**Figure 6:**
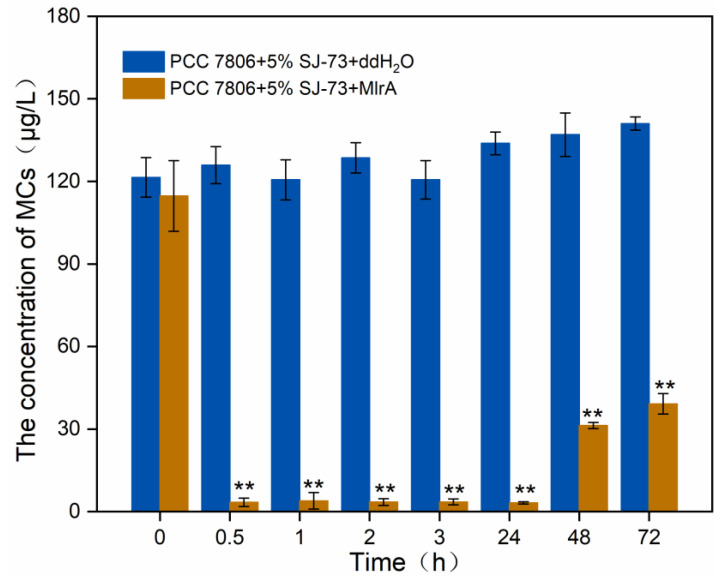
Changes on the concentration of extracellular MCs in *M. aeruginosa* PCC 7806 culture treated with 5% bacteria SJ-73 filtrates and 0.8 mg/L MlrA. Bars represent the standard errors of the means for triplicate. Significant differences are shown by asterisks: **, *p* < 0.01.

To acquire the optimal application conditions of MlrA to eliminate MCs caused by algicidal bacteria lysis, a preliminary experiment was performed to test the MlrA activity in SJ-73 treated unicellular *M. aeruginosa* cultures. MlrA at 0.8 mg/L was added to an *M. aeruginosa* PCC7806 culture that had been treated with 5% (*v*/*v*) SJ-73 filtrate for 3 days. As shown in Figure 6, the MC content decreased sharply from 125.89 μg/L to 3.39 μg/L after the addition of MlrA for 0.5 h, a decline of almost 97.30%, suggesting the quick elimination of MCs. The MC content remained consistently low until 48 h, before it increased slightly to 39.16 μg/L, indicating that the MlrA activity had decreased and was insufficient to degrade the persistently released MCs from incompletely cracked cells. Thus, a second dose of MlrA supplementation was necessary as the bacterial lysis of algae was relatively mild and the MlrA could not remain stable under 25 °C for a long period of time. 

#### 3.4.2. The Efficiency of MC Degradation by MlrA Following the Treatment of Colonial *M. aeruginosa* TH1701 Culture with SJ-73

**Figure 7:**
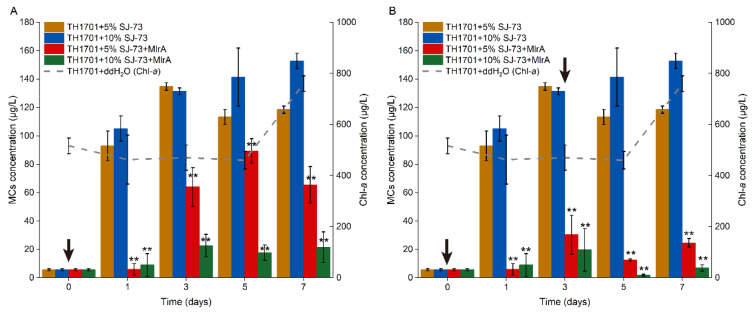
Changes on extracellular MCs concentration in colonial *M. aeruginosa* TH1701 culture treated with bacteria SJ-73. Black arrows represent the the day for adding MlrA in algal culture. The (**A**) MlrA was added into algal culture at the beginning of the experiment, and (**B**) the MlrA was added into algal culture at the beginning of the experiment and on the third day. Bars represent the standard errors of the means for triplicate. Significant differences are shown by asterisks: **, *p* < 0.01.

The efficiency of supplementation of MlrA following the treatment of colonial *M. aeruginosa* TH1701 culture with SJ-73 filtrate (5% and 10%, *v*/*v*) was tested. The MC concentration increased drastically from 5.61 μg/L to 118.49 μg/L and 152.841μg/L, respectively, after treatment with 5% and 10% (*v*/*v*) SJ-73 filtrate for one day. The MCs content were trending higher in the following days, achieving 21.16 and 27.29-fold increases compared with the initial content at day 7 (Figure 7A,B). As shown in Figure 7A, MlrA was only added once at day 0, after which the MC level decreased immediately to below 15.00 μg/L, and then increased slightly at day 3, indicating that a second MlrA supplementation was needed. As shown in Figure 7B, when MlrA was added twice, on day 0 and day 3, the MCs remained at a low concentration, ranging from 2.55 μg/L to 30.25 μg/L, with the peak value being observed on day 3 in both MlrA treatment groups. After the second MlrA addition at day 3, the MCs immediately decreased again. The MCs removal rates were 79.11% and 94.2% in the 5% and 10% (*v*/*v*) SJ-73 filtrate treatment groups, respectively, at day 7, suggesting that the introduction of two supplementations of MlrA enzyme and a higher dose of algicidal bacteria (10%, *v*/*v*) produced a better performance in removing MCs. 

## 4. Discussion

Algae-lysing bacteria are considered to be an effective and eco-friendly method to control the prevalence of cyanobacterial blooms. The selection of algae-lysing bacteria should consider the source and prey spectrum, to avoid exotic species invasion, the introduction of exogenous hazards, and detrimental effects on non-targeted organisms [41]. Isolation of the indigenous algicidal bacteria is the best option in a practical water body bioremediation process. In the present study, an indigenous algicidal bacterium, *Paenibacillus amylolyticus* strain SJ-73, was isolated from the sediments sampled from Taihu Lake. The highest algicidal rate of SJ-73 reached 93.88%. *Microcystis* blooms generally occur in the colony form and often coexist with several other cyanobacterial species under natural conditions; however, previous laboratory research mainly focused on the effect of water treatment processes on single *Microcystis* cells only. In this study, strong algicidal activity of *Paenibacillus* sp. SJ-73 against the major bloom-forming species, including colonial *Microcystis* and filamentous *Aphanizomenon* and *Anabaena*, was observed. Moreover, SJ-73 was also demonstrated to have no adverse impact on green algae. These results showed the promising potential of strain SJ-73 in controlling multi-species cyanobacterial blooms with a negligible influence on green algae. 

Our study further confirmed that the algicidal mechanism of strain SJ-73 was via secreting extracellular algicidal compounds that could cause an inhibition of photosynthesis and the disruption of cell membranes. Bioactive compounds, e.g., fusaricidin and polymyxin produced by *Paenibacillus* spp., were documented to have algicidal properties [42,43]. Although the algicidal substances were not characterized in this study, the three gene clusters involved in the synthesis of the antibiotics, polymyxin, bottromycin, and xenocoumacin, were predicted in the whole genome of *Paenibacillus* sp. SJ-73 using antiSMASH (https://antismash.secondarymetabolites.org) (data not shown). Moreover, a group of glycoside hydrolase (GH) family genes, including those encoding galactose hydrolase, xylosidase, α-mannasidase, and glucosidase, were also found in the SJ-73 genome based on the carbohydrate-active enzymes database (CAZyme) [44], which might play a role in decomposing the exopolysaccharides of colonial *Microcystis* (data not shown). This information provides a hint regarding the algicidal mechanisms of strain SJ-73.

The MC degradation process mediated by wild bacteria always show low efficiency and requires an adaptation period [45,46]. By contrast, heterologous expression of MlrA could greatly enhance the yields and the biodegradation abilities of MCs, as the MlrA activity of the cell-free extract from heterologous expression was 6800 times greater than that from the natural expression [32]. In our study, the degradation rate of [D-Asp^3^]MC-LR by crude *Sphingopyxis* sp. HW MlrA (cell-free extract from recombinant *E. coli*) was 17.82 mg L^−1^ h^−1^, whereas the value of MC-LR degradation by native *Sphingopyxis* sp. HW was 0.34 mg L^−1^ h^−1^ [47]. Further improvement of MlrA activity could be achieved by substituting a more efficient expression strain, e.g., *E. coli* C4 (DE3), as the host [48], or using an N-terminal maltose binding protein (MBP) tag, to increase enzyme solubility, stability, and overexpression [49]. 

The pH and temperature of environmental water bodies fluctuate frequently during the cyanobacterial bloom period; however, these two parameters are crucial for MlrA activity [32,46,48,49]. In this study, the degradation activities of heterologously expressed MlrA were tested under different pH and temperature conditions, revealing the optimal conditions for MC degradation as 30 °C and pH 8.0, which were slightly different to those determined by Dziga [48] (i.e., pH 7.5 and 20 °C). The degradative activities of MlrA were above 60% in the pH range of 6–9 and were maintained above 55% in the temperature range from 10 to 35 °C. These features will enhance the application potential for MlrA in field conditions. Except for pH and temperature, metalloprotease inhibitors (e.g., o-phenantroline and EDTA) should also be considered because MlrA is a metalloprotease [32].

Immobilized enzymes have advantages in recycling use and maintaining enzyme activity for a longer time in comparison to free enzymes, and the enzymatic activity of immobilized enzyme is strongly linked to the carrier materials. Excellent MC degrading activity of recombinant cells (BL21_MlrA), immobilized in alginate beads, has been reported [22,50]. Wu [51] prepared CysGO-immobilized MlrA and found that the degradation rate of CysGO-MlrA was slightly lower than that of the free enzyme, and that the degradation efficiency of the enzyme was maintained at >81% of the initial efficiency after 7 cycles of repeated usage. The immobilization of MlrA in alginate beads was attempted in the present study; however, the degradation efficiency of the immobilized MlrA was lower than that of free MlrA. Screening of more immobilization carriers is needed to improve the MlrA utilization efficiency in the future.

Normally, the treatment for toxic cyanobacteria is accompanied by the release of cyanotoxins, increasing the exposure risks for aquatic organisms and humans. A laboratory experiment conducted by Dziga [52] first proposed the combined treatment of *M. aeruginosa* with hydrogen peroxide (H_2_O_2_) and MCs biodegradation agents (*Sphingomonas* sp. or heterologously expressed MlrA enzyme), which eliminated both *Microcystis aeruginosa* and MCs simultaneously. In addition, the degradation activity of *Sphingomonas* sp. or the MlrA enzyme was not affected by H_2_O_2_ at a working concentration of 50 mg/L. Soon afterwards, this strategy was extended to an in situ mesocosm experiment in a eutrophic shallow lake (Lake Ludoš, Serbia) [48]. However, H_2_O_2_, as a safe algaecide, remains questionable because H_2_O_2_ was proved to upregulate MC production at concentrations of 2–3 mg/L [48]. In this study, we first proposed the co-application of algicidal bacteria and heterologous expressed MlrA enzyme, which could remove 92.07% of the *Microcystis* biomass and 79.11% of the MCs by treatment with 5% (*v*/*v*) fermentation filtrate for 7 days. In a preliminary experiment testing the activity on the axenic strain *M. aeruginosa* PCC 7806, after the supplementation with MlrA, the MC content in the treatment group decreased dramatically and remained at low concentrations. However, after 48 h the MC content began to increase slightly, suggesting the persistent releasing of MCs from incompletely cracked cells and the inactivity of MlrA after 2 days, thus a second supplementation was necessary. In the colonial *M. aeruginosa* TH1701 group, the introduction of a higher dose of algicidal bacteria (10%, *v*/*v*), and the supplementation with MlrA twice, achieved higher removal efficiencies of both algal biomass and MCs. Notably, although the minimum degradable MC concentration was below 0.8 μg/L, the MCs could not be completely removed, which might have been caused by the pH variation in the culture. As shown in Figure S4, in the 7806-MlrA group, the pH value increased from 8.6 to 9.5, while the pH value of TH1701-MlrA group remarkably decreased from 8.5 to 6.5; however, both shifts in pH could result in a decrease of MlrA activity (Figure S2). 

## 5. Conclusions

*Microcystis aeruginosa* blooms and MCs are causing serious environmental and ecological issues. The co-application of algicidal bacteria and MlrA enzymes to remove *M. aeruginosa* and MCs simultaneously was performed for the first time in this study, and the following conclusion might be formulated: (a) algicidal strain SJ-73 exhibited strong algicidal activities against colonial *Microcystis* as well as some other cyanobacterial bloom species; (b) the heterologously expressed MlrA enzyme showed high MC degradation efficiency, with a minimum degradable MC concentration below the safety threshold, and MlrA possessed a wide tolerance to pH (6.0–9.0) and temperature (10–35 °C); and (c) the marked release of MCs following cell rupture could be removed by supplementation with the MlrA enzyme; and a dose of 10% algicidal bacteria and two supplementations of MlrA enzyme exhibited the best performance. The outcome will provide valuable information for the design of strategies to deal with algae-contaminated water.

## Figures and Tables

**Table 1:** The algicidal efficiency of *Peanibacillus* sp. SJ-73 on different algae species.

Species	Algicidal Efficiency ^a^
*Microcystis aeruginosa* (PCC 7806)	83.97% ± 1.60
*Anabaena* sp. (PCC 7120)	71.35% ± 4.13
*Aphanizomenon flos-aquae* (FACHB 1168)	71.04% ± 6.20
*Chlorella sorokiniana* (FACHB 26)	3.67% ± 7.60
*Tetradesmus obliquus* (FACHB 416)	8.03% ± 7.00

^a^ The algicidal efficiency of SJ-73 against different algae species after 7 days’ treatment. The data are presented as mean ± standard deviations for triplicate.

## Data Availability

The nucleotide sequences of 16S rDNA gene from *Paenibacillus* sp. SJ-73 and *mlrA* gene from *Sphingopyxis* sp. HW in this study are available under the NCBI’s Genbank accession MZ182357.1 and MZ198825.1.

## Supplementary Materials

The following are available online at https://www.mdpi.com/article/10.3390/microorganisms9081594/s1, Figure S1: Phylogenetic tree based on bacterial 16S rDNA sequence of the isolated algicidal bacterium SJ-73 and closely related members. Figure S2: Influences of reaction pH (A) and temperature (B) on the activity of MlrA. Figure S3: The relative activity of the immobilized and free enzyme at different storage temperature (4 °C and 25 °C). Figure S4: variations of pH in *M. aeruginosa* PCC 7806 (A) and colonial *M. aeruginosa* TH1701 (B) cultures treated with bacterium SJ-73 filtrate for 7 days.
