# Supplementary material for: Simultaneous Removal of the Freshwater Bloom-Forming Cyanobacterium Microcystis and Cyanotoxin Microcystins via Combined Use of Algicidal Bacterial Filtrate and the Microcystin-Degrading Enzymatic Agent, MlrA
Source: Microorganisms. 2021 Jul 27;9(8):1594. doi: 10.3390/microorganisms9081594 (PMC8401626; doi:10.3390/microorganisms9081594)
Supplement: Supplementary file 1 [file microorganisms-09-01594-s001.zip › microorganisms-1298743-supplementary.pdf]

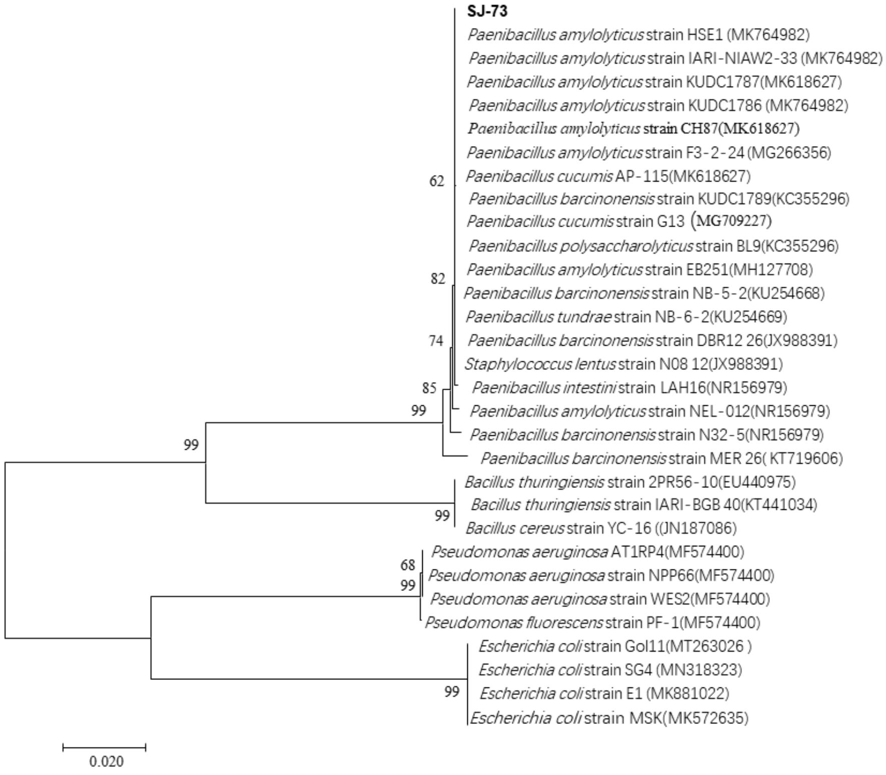

Figure S1. Phylogenetic tree based on bacterial 16S rDNA sequence of the isolated algicidal bacterium SJ-73 and closely related members. Tree topology shown is from Neighbor-joining analysis. Scale bar represents 0.020 nucleotide substitution per position.

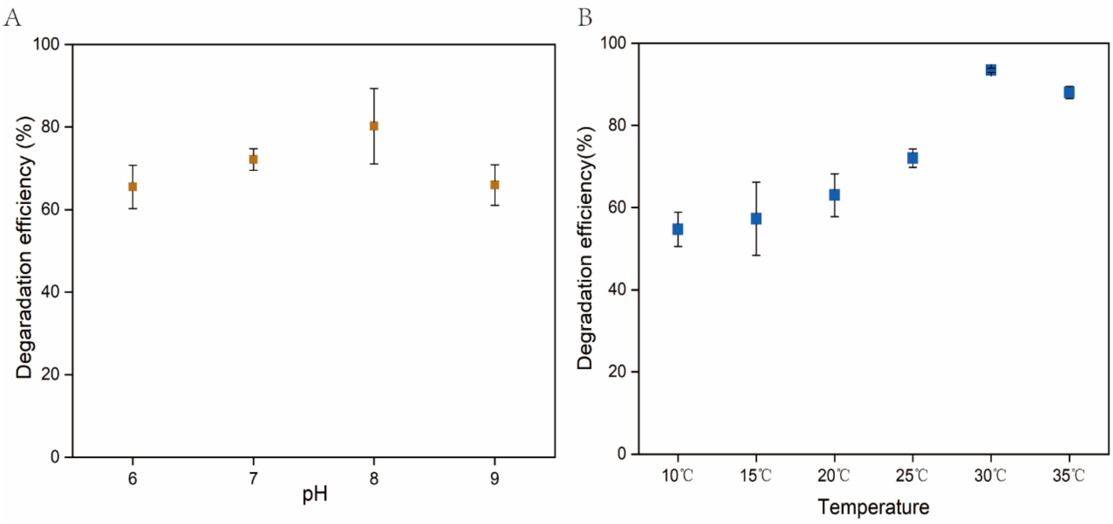

Figure S2. Influences of reaction pH (A) and temperature (B) on the activity of MlrA.

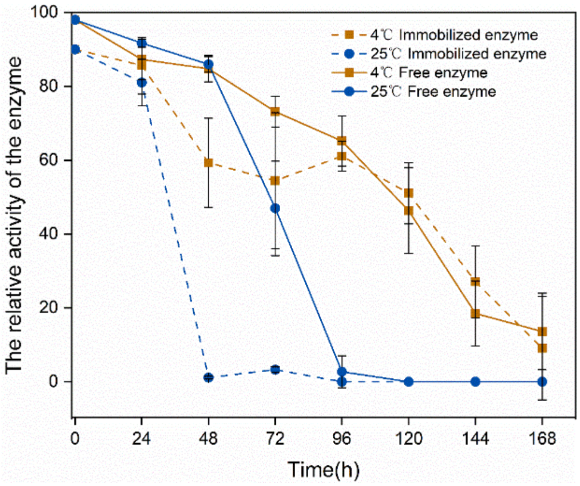

Figure S3. The relative activity of the immobilized and free enzyme at different storage temperature (4°C and 25°C). Bars represent the standard errors of the means for triplicate.

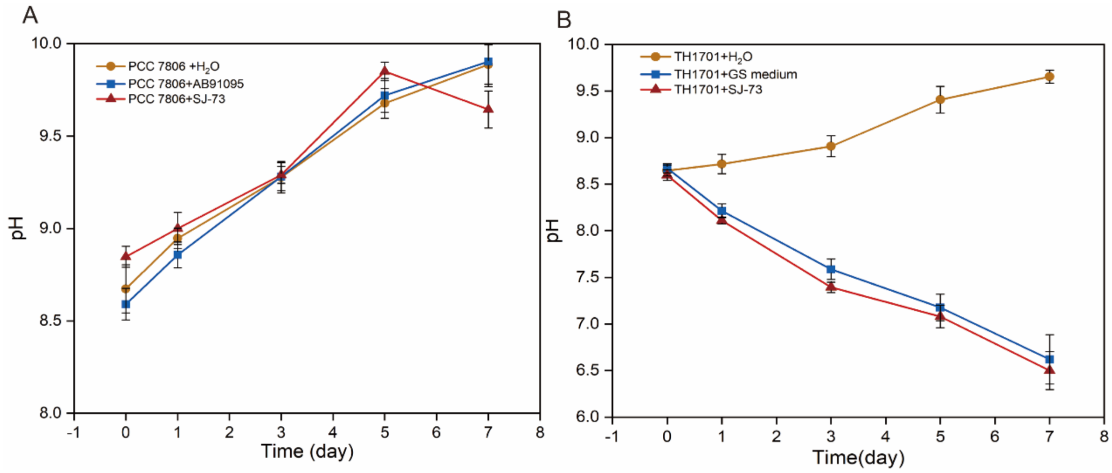

Figure S4. Variations of pH in *M. aeruginosa* PCC 7806 (A) and colonial *M. aeruginosa* TH1701 (B) cultures treated with bacterium SJ-73 filtrates for 7 days.
